# Ninth International Medical Congress, Washington, D. C., September, 1887

**Published:** 1888-01

**Authors:** 


### NINTH INTERNATIONAL MEDICAL CONGRESS. WASHINGTON, D. C., SEPTEMBER, 1887.

### SECTION XVIII, DENTAL AND ORAL SURGERY.

Reported for the Independent Practitioner, by "Mrs. M. W. J."

Continued From Page 647, Vol. VIII.

### WEDNESDAY MORNING SESSION.

The opening paper was from Dr. Pradère de Moine, Lyons, France, entitled, "La Phthisie Vaincue par la Permanence des Medicaments aux Milieu du Palais." (Phthisis Cured by the Continuous Application of Medicaments to the Palate).

A translation of portions only of the paper was read by the Secretary.

After a brief discussion, it was voted to refer the paper to the Section of Practical Medicine, as not coming within the sphere of the dental practitioner.

Dr. J. Von Metnitz, of Vienna, Austria, read a paper, entitled "Osteo Myelitis."

He said that inflammation of the medullary substance of the maxillaries was seldom met with outside of the hospitals, where it was probably aggravated by uncleanliness on the part of the patient. The original causes in these cases were not easily discovered. Dr. Metnitz then described two cases, in both of which the patient died. One was that of a man of forty-three years of age, for whom a number of teeth had been extracted. He was soon after taken with high fever; on the fifth day he was delirious; on the seventh day he was taken to the hospital. The stench was then intolerable, the left cheek and temporal region greatly swollen, the eye-ball distended and the jaws set. He died the next day. Examination showed both upper and lower alveoli empty, with the exception of the remains of the third molar with a socket six millimeters deep. There was necrosed bone in all directions, and the articulation was entirely destroyed. In the second case, a patient of seventeen years old, there was chronic inflammation of the lower maxilla, the periosteum was destroyed and there was no nutrition of the parts.

Dr. M. L. Rhein, of New York, opened the discussion of this paper. He thought the free use of the bur in removing all infiltrated bone would often produce beneficial results, if we go beyond the line of infiltration.

Dr. G. J. Friedrichs, of New Orleans, thought the opinion of the author of the paper, as to treatment, worth nothing, as both patients died. He had had no experience in cases causing death, but thought it might sometimes be necessary to excise the entire lower maxilla to effect a cure.

The paper passed without further discussion.

Dr. M. G. Jenison, of Minneapolis, read a paper, entitled "Art in Dentistry."

He said that the perfection of talent lay in the application of the principles of art to practice, for which dentistry opens the widest field. In restoring teeth to beauty and usefulness it must be borne in mind that there was a definite purpose in every line, in every form, in every surface; to mar or to cut away anything is to destroy that purpose. A very slight loss will change both articulation and expression of features. The profile of Apollo is the type of physical beauty, but the slightest modification of any feature would detract materially from that beauty. The face of a patient must be made the subject of careful study. The play of the muscles of the mouth must be watched, when open, when closed, when in full action, when in repose. In the arrangement of the teeth there are great opportunities; the teeth should be selected with reference to both temperament and age; gum sections afford no scope for originality.

The discussion of this paper was opened by Dr. John Allen, of New York. He spoke of the antiquity of dental operations, as shown in the tombs of prehistoric races, in Egyptian mummies, in the works of Greek and Latin poets, of medical authors from Galen down; but it is only in the present century that elaborate works on dentistry have been published, and that we have dental journals, dental colleges, dental associations. With these facilities dentistry now stands higher than ever before; yet there are greater heights to be attained, especially in the prosthetic department. Dr. Allen disclaimed the term "mechanical dentistry." The restoration of form and feature, of expression to the mouth and face, is not mechanical work; it is art, and required the highest degree of artistic skill. A certain degree of knowledge can be acquired through books, but for the higher practical points a man must depend on his own individual powers of conception. Such efforts are the means of advancing dental science. The knowledge that is conquered by labor is truly our own. Through individual efforts our profession will advance, nourished by the dew of wisdom, warmed by the sun of science.

The subject was passed without further discussion.

### WEDNESDAY AFTERNOON SESSION.

The session was held in the National Theatre, which was darkened to allow of stereopticon illustrations of a paper on "The Origin of the Dental Fibril," by Dr. R. R. Andrews, of Cambridge, Mass., and one on "Protective Dentine, or Dentine of Repair," by Dr. M. H. Fletcher, of Cincinnati, Ohio.

There was also an exhibit of Photo-Micrographs, by Dr. J. Howard Mummery, of London, England. (The auditorium being darkened, only very inadequate notes could be taken by the reporter.)

Dr. Andrews spoke of the different processes adopted by different investigators in their efforts to get at the exact structure and the minute details of the process of dentification. When it is possible to work nearer life we may hope to reach more satisfactory results. The specimens used by Dr. Andrews were taken from the embryo, at about the time of birth, and immersed in a preparation of osmic acid, which was changed daily for two or three days. Instead of immersing them in alcohol, they are dried on bibulous paper and then placed in melted paraffine or lard, which is left to cool. Very thin sections can be cut, and we work as near life as is possible with our present knowledge.

The illustrations thrown upon the screen were guaranteed by the author to be actual reproductions of tissue, not a line having been added to carry out a theory, and all the work done by himself, from the selection of specimens to mounting the photo-slides.

The successive illustrations showed the gradual development of the tooth from the first dipping down of a line of epithelium (from the jaw of an embryo pig two inches in length), through its enlargement and change to flask-shape; the formed papilla below the enamel-cap, gradually assuming the tooth shape, with the permanent tooth-germ budding from the side; the growth of the pulp within the dental sacculus, and the cement organ; the beginning of calcification with the odontoblasts, and fibrils forming. The author laid great stress on the difference in outline of the square abrupt-edged dentine cells, and the fibril-cells in elongated pear-shape, with fibrils drawn out when broken off in cutting the sections. The last of the series was a section through the jaw bone showing all the tissues, dentine and enamel, with wavy lines showing anastomosis between the two.

Dr. Frank Abbott, of New York, opened the discussion of Dr. Andrews' paper.

He said that the stereopticon views so far exceeded his anticipations that he hardly knew what to say. The profession owed Dr. Andrews a great debt for the patience, earnestness and intelligence with which he had worked for the advancement of his brethren. He must, however, differ from his view of the origin of the dental fibril. The papilla is a mass of myxomatous tissue, liberally supplied with medullary elements. The medullary corpuscles coalesce and form the odontoblasts about the same time that the enamel organ is observed forming the enamel-cap upon the papilla. The odontoblasts, when viewed under high power, show a delicate reticulum, which unites the nuclei with the walls of each corpuscle and with each other. This reticulum, as well as the walls of the odontoblasts, is the living matter which remains as the living portion of the dentine. As the calcareous basis-substance is deposited, a certain territory of the papilla becomes dentine, another row of odontoblasts making its appearance, from the ends and sides of which prolongations of the living matter may be seen running into the canaliculi of the dentine already formed. The pear-shaped odontoblast gives off one, while the broad or square shapes may give off several prolongations. If only the pear-shaped odontoblasts gave off the fibrils, territories of considerable size would be left in the dentine with no canaliculi, or any provision for furnishing these territories with living tissue. To account for the origin of the dental fibril, it is not necessary to have any special shaped cells wedged in between the others.

Dr. M. H. Fletcher, of Cincinnati, Ohio, gave a series of stereopticon views of "Protective Dentine, or Dentine of Repair;" dentine thrown out by the pulp to protect itself from external injuries or from exposure. It differs from normal dentine in being more horny or translucent, and in the comparative irregularity of the tubuli. It is often yellow or brown in color; it is built up more rapidly than normal dentine, and is also softer. It is due to some disturbance of the pulp, through the fibrils, stimulating it to abnormal efforts. The pulp sometimes dies, exhausted through its own efforts, leaving unoccupied space, or the pulp is so contracted that it dies from that cause, or pulp nodules may be formed. There is seldom complete calcification of the pulp-chamber, as the pulp must have room in which to perform its functions. With the death of the pulp, putrid matter forms an abscess.

The views exhibited the process of formation of protective dentine in cases of abrasion, in cavities of decay approaching the pulp-chamber, pulp nodules, deposits on the cementum, roots of teeth cemented together, etc.

The discussion of this paper was opened by Dr. W. X. Sudduth.

He said that this secondary dentine was developed by odontoblasts retained for that purpose, remaining quiescent on the surface of the pulp, but which, when necessary, are stimulated to renewal of function.

Dr. Sudduth said that Dr. Andrews had laid great stress on the form of the fibril-producing odontoblasts—the pear-shaped cells—but though his illustrations were the finest seen, they showed more than he himself saw, for though some were spindle-shaped, some square, some curved, there were fibrils projecting from all alike, and from two to half a dozen from some. If the odontoblasts are crowded they may be triangular or wedge-shape. Stress should be laid on function, not on form.

Dr. J. Howard Mummery, of London, England, exhibited a series of "Photo-Micrographs of Tooth Structure," explaining his method of producing them, and the mode of bringing out the colors, etc.

Dr. Taft congratulated the members on the rich feast they had enjoyed.

THURSDAY MORNING SESSION.

Dr. L. C. Ingersoll, of Keokuk, Iowa, read a paper entitled "Inflammatory Processes," and not "Inflammation of the Oral Tissues," as announced on the programme. He would not treat of the etiology or bacteriology of inflammation, but only of its pathology, as being the most important feature to the dentist, causing much of the suffering they are called upon to relieve. In every case of inflammation of the tissues there is something in the environment which is not in harmony, and which causes a deviation from the physiological to the abnormal, causing a reversion or retrograde action. In all nature we see the operation of dual forces; in electricity we call it attraction and repulsion; in physics it is the positive and negative; so abnormal physiology is pathology. The processes of inflammation were given in diagram, as follows:

| | | | | | |
|---|---|---|---|---|---|
| Irritation. | Inflammation, Acute. | Congestion. | Necrosis. | Exfoliation. | Somatic Death. |
| | | | Gangrene. | Slough. | |
| | | | Suppuration. | Molecular Death. | |
| | | Chronic Inflammation. | Hypertrophy | | |
| | | | Tumefaction. | | |
| | | | Induration. | | |

Dr. Ingersoll read only portions of his paper. Passing over the first stage, irritation, he dwelt somewhat at length upon acute inflammation and congestion, the short, quick route to death of the tissues, chronic inflammation being the longer road, or "main line," with "stop-over stations," as hypertrophy, or tumefaction, or induration, Congestion, he said, was the most alarming feature or stage of inflammation, being next door to death of the tissues, through necrosis, gangrene, suppuration—necrosis ending in exfoliation, gangrene in slough, suppuration being molecular death. An illustration showed the result of congestion in lessened vascular action, impaired flow of blood, with the massed red corpuscles clogging up the canal, the white corpuscles imbedded in the walls and percolating into the surrounding tissues, red corpuscles following, the debris constituting pus. As this work of destruction goes on, a work of reconstruction is also instituted, the leucocytes when breaking through walls meeting hordes of embryonic tissue-cells at work repairing; but for this work of construction going on simultaneously, speedy death would intervene; an alveolar abscess would mean, not merely swelling and pain, but would be a death stroke. Inflammation in cancellous bone and in dentine he described as similar processes, the swelling of the medullary tissues being at the expense of the cervical walls of the canaliculi or tubuli, which are thus softened and broken down. When dentine is keenly sensitive it is evidence of inflammation of the dentinal fibrils communicating with the pulp. Another illustration showed the pulp as a central organ with radiating processes—a nerve-centre, which should be called the dental ganglion; the pulp being an inert homogeneous mass, we might as well call the brain the cranial pulp.

The discussion of this paper was assigned to Dr. A. O. Rawls, of Lexington Kentucky, who characterized the paper as a *résumé* of what is contained in books, many portions being almost taken verbatim from Connheim and Stricker. He did not think that Dr. Ingersoll's view of the part taken by embryonic tissue-cells in connection with leucocytes would be sustained. He thought Dr. Ingersoll's illustration of a pulp-tumor rather that of fungous growth. Sensitive dentine he considered the result of a hyperæmic condition of the fluids of the tooth-packing into the tubules an additional amount of fluid, the increased flow of blood causing a congested condition; but there was no inherent sensitiveness. Pressure of the pulp gives pain through compression, not through any inherent sensitiveness of the pulp or of the fibrils; if pressed laterally, where it can give way, the pressure does not cause pain. Dentine, also, is broken down and absorbed under compression, with rapid flow of the watery parts of the blood in the tubuli, but not because of any special inflammatory action in the tooth.

Dr. Fletcher, of Cincinnati, related some experiments with electricity on the mesentery of a frog. The leucocytes gathered along the edge of the capillaries till they were completely occluded, when migration began; turning on the Farradic current, their course was changed; those that were passing through died *en route* and circulation was re-established, seeming to show that the life of the corpuscle is affected by electricity or other irritating applications.

After some further discussion of the paper by Drs. Storey, of Texas, and Rhein, of New York, Dr. Ingersoll appealed to the judgment of the Section after reading the entire paper when published, just criticism being impossible from the scattered fragments he had read.

Dr. C. L. Goddard, of San Francisco, Cal., read a paper, entitled "Pain in the Temporo-Maxillary Joints, Caused by Irregularity of the Teeth." This was the record of a case in which the patient suffered severe pain during mastication, even of a crust of bread, but not at other times. An impacted *dens sapientiæ* was at first suspected, but they were found all in place. Examination showed that the inferior maxilla was much longer than the other. The mucous membrane was in normal condition, with no tenderness to pressure anywhere. Examination of the articulation showed that when the jaws closed normally, only the cutting edges of the incisors articulated, the bicuspids and molars not antagonizing by one-sixteenth of an inch. In mastication it was necessary to protrude the inferior incisors beyond the superior to articulate the bicuspids and molars. Dr. Goddard, being convinced that the pain was due to the continuous strain on the muscles and ligaments, experimented on himself, showing that a great effort was necessary to bite hard with the jaw protruding. It was, therefore, necessary to change the articulation and allow of normal articulation. This was effected by means of a Coffin split-plate, and in a few weeks the superior incisors closed over the inferior, and normal articulation was secured, curing the long-standing pain.

There was no discussion of this paper.

Dr. E. S. Chisholm, of Tuscaloosa, Ala., read by title a paper on "Influence of Weather Changes on the Human Organism." He said that he regretted that time did not permit the reading of his paper, as he was convinced the subject was one of great practical importance, and one in which Government aid and coöperation were desirable.

His views on the subject were the results of fifteen years' daily observation of the effects of the variations of atmospheric pressure on the human organism, its tissues and nerves. As a practical point, he could foretell almost unfailingly from the mercurial barometer the general class of dental suffering he will meet during the day.

President Taft said that for six or eight years he had carried a barometer, having commenced observations at the suggestion of Dr. Chisholm, and could testify to the value of his views, and regretted that the paper could not be heard.

(TO BE CONTINUED.)

